# Rifampicin tolerance and growth fitness among isoniazid-resistant clinical *Mycobacterium tuberculosis* isolates: an in-vitro longitudinal study

**DOI:** 10.1101/2023.11.22.568240

**Published:** 2023-11-22

**Authors:** Srinivasan Vijay, Nguyen Le Hoai Bao, Dao Nguyen Vinh, Le Thanh Hoang Nhat, Do Dang Anh Thu, Nguyen Le Quang, Le Pham Tien Trieu, Hoang Ngoc Nhung, Vu Thi Ngoc Ha, Phan Vuong Khac Thai, Dang Thi Minh Ha, Nguyen Huu Lan, Maxine Caws, Guy E. Thwaites, Babak Javid, Nguyen Thuy Thuong Thuong

**Affiliations:** a Oxford University Clinical Research Unit, Ho Chi Minh City, Vietnam; b Centre for Tropical Medicine and Global Health, Nuffield Department of Medicine, University of Oxford, Oxford, United Kingdom; c Theoretical Microbial Ecology, Institute of Microbiology, Faculty of Biological Sciences, Friedrich Schiller University, Jena, Germany; d Cluster of Excellence Balance of the Microverse, Friedrich Schiller University Jena, Jena, Germany; e Department of Clinical Sciences, Liverpool School of Tropical Medicine, Liverpool, United Kingdom; f Division of Experimental Medicine, University of California, San Francisco, California, USA; g Pham Ngoc Thach Hospital, Ho Chi Minh City, Vietnam

## Abstract

Antibiotic tolerance in *Mycobacterium tuberculosis* leads to less effective bacterial killing, poor treatment responses and resistant emergence. There is limited understanding of antibiotic tolerance in clinical isolates of *M. tuberculosis*. Therefore, we investigated the rifampicin tolerance of *M. tuberculosis* isolates, with or without pre-existing isoniazid-resistance. *In-vitro* rifampicin survival fractions determined by minimum duration of killing assay in isoniazid susceptible (n=119) and resistant (n=84) *M. tuberculosis* isolates. Rifampicin tolerance was correlated with bacterial growth, rifampicin minimum inhibitory concentrations (MICs) and isoniazid-resistant mutations. The longitudinal isoniazid-resistant isolates were analyzed for rifampicin tolerance based on collection time from patients and associated emergence of genetic variants. The median duration of rifampicin exposure reducing the *M. tuberculosis* surviving fraction by 90% (minimum duration of killing-MDK90) increased from 1.23 (95%CI 1.11; 1.37) and 1.31 (95%CI 1.14; 1.48) to 2.55 (95%CI 2.04; 2.97) and 1.98 (95%CI 1.69; 2.56) days, for IS and IR respectively, during 15 to 60 days of incubation respectively. Increase in MDK90 time indicated the presence of fast and slow growing tolerant sub-populations. A range of 6 log_10_-fold survival fraction enabled classification of tolerance as low, medium or high and revealed isoniazid-resistance association with increased tolerance with faster growth (OR=2.68 for low vs. medium, OR=4.42 for low vs. high, *P*-trend=0.0003). The high tolerance in longitudinal isoniazid-resistant isolates was specific to those collected during rifampicin treatment in patients and associated with bacterial genetic microvariants. Our study identifies a range of rifampicin tolerance and reveals that isoniazid resistance is associated with higher tolerance with growth fitness. Furthermore, rifampicin treatment may select isoniazid-resistant isolate microvariants with higher rifampicin tolerance, with survival potential similar to multi-drug resistant isolates. These findings suggest that isoniazid-resistant tuberculosis needs to be evaluated for rifampicin tolerance or needs further improvement in treatment regimen.

It is made available under a CC-BY 4.0 International license.

## Introduction

*Mycobacterium tuberculosis* causes around 10 million cases of tuberculosis (TB) each year and 1.5 million deaths^1^. Challenges to successful TB treatment include bacterial evolution and diversification under host stresses and antibiotics, leading to differential antibiotic susceptibility even among genetically-susceptible *M. tuberculosis* isolates^2^. Based on killing dynamics, the differential susceptibility can be classified into two, 1) antibiotic tolerance observed as reduced rate of killing of the entire bacterial population, and 2) antibiotic persistence observed as reduced rate of killing of sub-populations compared to more susceptible bacteria^3^. There are differences in the molecular mechanisms of tolerance and persistence, for example stress conditions inhibiting bacterial protein or ATP synthesis can result in population level tolerance^4^, whereas stochastic expression and induction of bacterial stress response result in persistent subpopulations^5^. Clinically susceptible isolates exposed to host stresses and antibiotic selection can exhibit increased antibiotic tolerance and persistence^6–8^, supporting this studies have shown emergence of mutations increasing tolerance or persistence among clinical *M. tuberculosis* isolates^9–12^.

Emergence of rifampicin tolerance or persistence, a key drug in TB treatment is a major concern considering the emergence of multi-drug resistant (MDR, resistant to at least isoniazid and rifampicin) tuberculosis^13^. Several mechanisms lead to rifampicin tolerance, heteroresistance or persistence^14^. These include efflux pump overexpression^15^, mistranslation^16^, overexpression of rifampicin target *rpoB*^17^, cell size heterogeneity^18–20^ and the redox heterogeneity in bacteria^21^.

Recent study has implicated the antibiotic tolerance in clinical isolates as a risk factor for hard-to treat infections and tolerance can also contribute to the emergence of resistance^22^. One of the difficulties in reducing the duration of anti-tuberculosis treatment is that shorter regimens are associated with high rates of relapse of infection. Relapses are believed to be partly due to hard-to treat bacterial phenotypes^23^. Therefore, it is important to identify hard-to treat phenotypes and stratify the treatment regimens based on the risk factors for poor treatment responses^23^.

Apart from antibiotic susceptibility variations, another concern in standard TB treatment is the emergence of resistance to isoniazid (IR). There is globally around 10% prevalence of IR among clinical *M. tuberculosis* isolates^24^. IR is difficult to rapidly diagnose during drug susceptibility testing, and is associated with worse treatment outcomes compared to isoniazid-susceptible (IS) *M. tuberculosis* isolates^24^. Importantly, IR is also associated with subsequent emergence of rifampicin resistance leading to MDR TB^25^. Therefore, emergence of antibiotic tolerance or persistence among IS and IR clinical *M. tuberculosis* isolates may contribute to poor treatment response and the emergence of MDR-TB.

In addition to antibiotic survival, emergence of growth heterogeneity post-stress in bacterial survival fractions can lead to trade-offs between growth fitness and population survival, with the fast-growing sub-population mainly contributing to growth and the slow growing sub-population as a reservoir strategy for survival under future stresses^26^. Rifampicin treatment can result in differentially detectable sub-populations of *M. tuberculosis*, which can grow only in liquid medium as compared to solid medium^27^. Therefore, in determining risk of post-treatment relapse, it is important to consider, alongside tolerance range, the degree of growth heterogeneity within tolerant subpopulations.

Despite its potential importance for the TB treatment, the distribution of antibiotic tolerance among clinical *M. tuberculosis* isolates is unknown, and routine clinical microbiology diagnosis does not include any assays for tolerance. The growth fitness of antibiotic tolerant subpopulations, and the association of pre-existing (other drug) resistance with antibiotic tolerance is completely unknown. Antibiotic tolerance and persistence can be differentiated by linear vs bi-phasic killing curves or single vs bi-modal growth rate distribution^5^. Another consideration is that tolerance can mask persistence,^5^ therefore a single assay may not determine both tolerance and persistence among clinical isolates^5^.

To address this knowledge gap, we developed a most-probable number (MPN) based minimum duration of killing (MDK) assay to determine the rifampicin tolerance among clinical *M. tuberculosis* isolates in a medium-throughput manner^28^. In the current study, we investigated the rifampicin tolerance in a large set of IS (n=119) and IR (n=84) clinical *M. tuberculosis* isolates and its correlation with bacterial growth rate, rifampicin MICs, IR-mutations and the rifampicin treatment selection in patients.

## Results

### Study design

**Figure 1.**
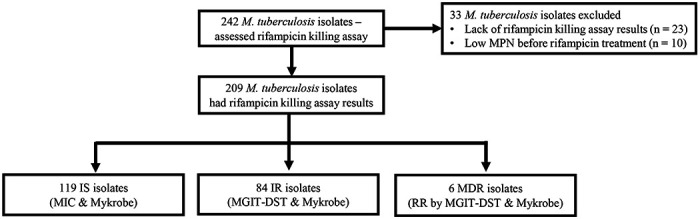
Study design. IS – Isoniazid susceptible, IR – Isoniazid resistant, RR – Rifampicin resistant

We investigated rifampicin tolerance and its association with isoniazid susceptibility among 242 clinical *M. tuberculosis* isolates. We treated susceptible isolates with rifampicin (2μg/mL), a concentration several times higher than their MICs (supplementary table 1), and at 0, 2 and 5 days determined fractional survival following 15 and 60 days of culture (figure 1). Higher survival fractions indicate higher rifampicin tolerance, and differences in survival fraction determined between 15 and 60 days of incubation indicated greater growth heterogeneity in rifampicin tolerant sub-populations. 23 of the isolates grew poorly in the absence of antibiotic, and a further 10 had low initial MPN, making accurate determination of survival fractions difficult (figure 1), and these 33 isolates were removed from further analysis. Of the remaining 209 isolates, 119 IS, 84 IR and 6 resistant to both rifampicin and isoniazid, MDR. The MDR isolates were controls and comparators as isolates with a known high degree of rifampicin tolerance^28^.

### Distribution of Rifampicin tolerance in IS and IR isolates

**Figure 2.**
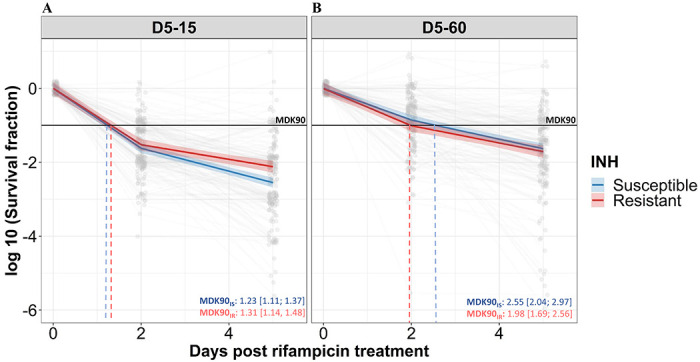
Rifampicin survival curve in isoniazid susceptible and resistant clinical *M. tuberculosis* isolates. **(A, B)** The bacterial kill curve as measured by log10 survival fraction from data collected at 0, 2 and 5 days of rifampicin treatment followed by incubation for 15 and 60 days respectively. Data from individual isolates are shown as the grey dots connected by lines. Estimated mean with 95% credible interval (bold coloured line and colour shaded area respectively) of isoniazid susceptible (IS – blue, n = 119, 117 for 15 and 60 days of incubation respectively) and resistant (IR – red, n = 84, 80 for 15 and 60 days of incubation respectively) clinical *M. tuberculosis* isolates based on linear mixed effect model implemented in a Bayesian framework. One log10 fold or 90% reduction in survival fraction is indicated (MDK90, black horizontal line) and the mean time duration required for 90% reduction in survival (MDK90, minimum duration of killing time) at 15 and 60 days of incubation is indicated by vertical dashed lines with respective colours for IS and IR isolates.

We analyzed the rifampicin survival fraction and the kill curve for IS and IR *M. tuberculosis* isolates, at 0, 2 and 5 days of rifampicin treatment followed by 15 and 60 days of incubation (figure 2). Following 5 days of rifampicin treatment, the average survival fraction reduced by 90-99% of the starting bacterial population (figure 2). Of note, the average time required for 90% survival fraction reduction (MDK90) was 1.23 (95%CI (Confidence interval) 1.11; 1.37) and 1.31 (95%CI 1.14; 1.48) days for IS and IR respectively when survivors were incubated for 15 days, but rose to 2.55 (95%CI 2.04; 2.97) and 1.98 (95%CI 1.69; 2.56) days for 60 days for IS and IR isolates respectively (figure 2). This shift in the MDK_90_ indicated the presence of growth heterogeneity within the tolerant subpopulation – with both fast and slow-growing bacteria within tolerant subpopulations. For most of the isolates MDK90 time could be calculated but other parameters of tolerance and persistence such as MDK_99_ (at 15 day=81% (170/209), 60 day=41% (86/209)) and MDK_99.99_ (at 15 day=11% (22/209), 60 day=8% (17/209)) could be calculated for only a fraction of 209 isolates and rest were beyond the assay limits (supplementary figure 1). Intriguingly, we observed a significant difference in rifampicin tolerance between IS and IR isolates – but only in the 15 days post recovery. The difference had disappeared by 60 days (figure 2). Therefore, we decided to consider survival fractions with 15 and 60 days recovery for further analysis, the earliest and latest time points for determining the fast-and slowly-growing rifampicin tolerant subpopulations.

### Isoniazid resistance is associated with fast-growing rifampicin tolerant subpopulations

**Figure 3.**
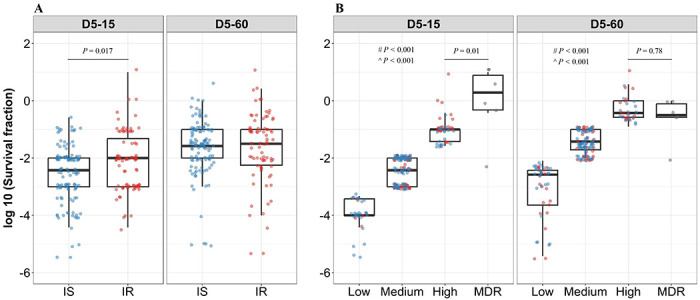
Rifampicin survival fraction distribution in isoniazid susceptible and resistant clinical *M. tuberculosis* isolates. (A) Log10 rifampicin survival fraction distribution, with median and IQR (inter quartile range), of individual isoniazid susceptible (IS, blue dots, n = 119, 117 for D5-15, and D5-60 respectively), and resistant (IR, red dots, n = 84, 80 for D5-15, D5-60 respectively) isolates for 5 days of rifampicin treatment as determined at 15 and 60 days of incubation (D5-15, D5-60 respectively). (B) Rifampicin tolerance distribution in both IS (blue dots) and IR (red dots) isolates combined together was used to group them as low (< 25th percentile, n = 33, 47 for D5-15, and D5-60 respectively ), medium (from 25th to 75th percentile, n = 124, 115 for D5-15, and D5-60 respectively) and high (above 75th percentile, n = 46, 35 for D5-15, and D5-60 respectively) level of rifampicin tolerance and compare it with rifampicin tolerance of MDR clinical *M. tuberculosis* isolates (grey dots, n = 6), after 5 days of rifampicin treatment and determined at 15 and 60 days of incubation (D5-15, D5-60 respectively). Statistical comparisons between Low, Medium, and High or MDR were made by using Wilcoxon rank-sum test. ^#^ - p-value for comparing the Low and High tolerance groups, ^ - p-value for comparing the medium and High tolerance groups.

To further investigate the relationship between rifampicin tolerance, growth fitness and isoniazid susceptibility, we compared fractional survival at 15 and 60 days recovery following 2 and 5 days of rifampicin treatment in IS (n=119) and IR (n=84) isolates (figure 3A, supplementary figure 2). There was no significant difference in rifampicin tolerance between IS and IR isolates at 2 days of treatment (supplementary figure 2). At 5 days of rifampicin treatment and both early and late recovery time points, IS and IR isolates showed a broad distribution of fractional survival-spanning 1 million-times difference in rifampicin susceptibility (figure 3A). At the 15 day recovery period, IR was significantly associated with higher survival to rifampicin treatment as compared to IS isolates (P=0.017, figure 3A), whereas at 60 days, fractional survival increased in both groups with no difference according to isoniazid susceptibility (figure 3A). These results suggest that the difference between IS and IR rifampicin tolerant subpopulations is within their fast-growing tolerant bacilli only.

To further refine distribution of rifampicin tolerance between isolates, fractional rifampicin survival was parsed as low, medium or high as defined by falling within the 25^th^, 75^th^ and 100^th^ percentiles of survival fractions following rifampicin treatment and either 15 or 60 days recovery (figure 3B). As expected, there was substantially lower tolerance to rifampicin in low and medium groups compared with MDR isolates. Surprisingly, tolerance to rifampicin between non-rifampicin resistant “high” tolerance strains and MDR strains was not significantly different (P=0.78, figure 3B), and these high tolerant strains were characterized in both IS and IR isolates. This suggests that within the IR, high tolerant subgroup, antibiotic susceptibility (to both rifampicin and isoniazid) may be similar to *bona fide* MDR strains. Analyzing rifampicin tolerance subgroups between IS and IR strains, at the early, 15 day recovery time-point, the majority (79%, 26/33) of “low” rifampicin tolerant strains were isoniazid susceptible. By contrast, IR isolates were over-represented in the “medium” and “high” tolerant subgroups (OR of 2.7 and 4.4 respectively–table 1). These associations disappeared with longer (60 day) recovery post antibiotic treatment, confirming that IR isolates harbored fast-growing, high-level rifampicin-tolerant bacilli compared with IS isolates (table 1).

### Association between rifampicin tolerance and relative growth in absence of antibiotics, rifampicin MICs, isoniazid resistant mutations of *M. tuberculosis* isolates

Clinical isolates of *M. tuberculosis* exhibit a large degree of lag time and growth heterogeneity^29^, as well as differences in rifampicin MICs or isoniazid-resistant mutations. Prior studies showed that slow growth rate and non-replicating persistence were correlated^30^, therefore we wished to investigate the association between growth rates in the absence of antibiotic treatment, rifampicin MIC distribution, isoniazid-resistant mutations and rifampicin tolerance distribution in *M. tuberculosis* isolates.

**Figure 4.**
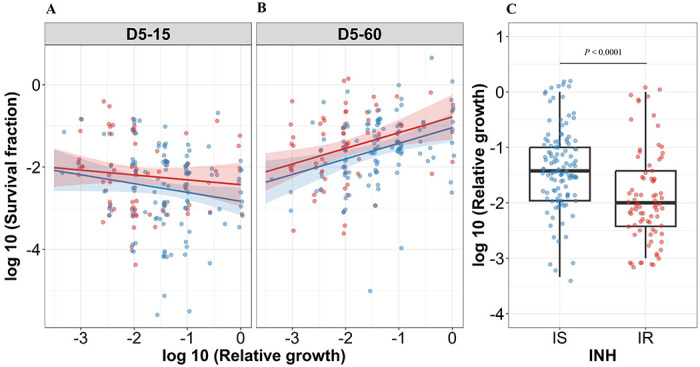
Correlating rifampicin survival fraction with before treatment relative growth of clinical *M. tuberculosis* isolates. Log10 survival fraction at 5 days of rifampicin treatment as determined at 15 days (A) and 60 days of incubation (B), for isoniazid susceptible (IS, blue dots) and resistant (IR, red dots) isolates respectively, correlated with the log10 relative growth determined before rifampicin treatment for clinical *M. tuberculosis* isolates. Coefficients of linear regression for (A) IS = −0.21 [−0.44, 0.007], *P* = 0.058; IR = −0.12 [−0.38, 0.14], *P* = 0.37, and (B) IS = 0.38 [0.15, 0.61], *P* = 0.0014; IR = 0.38 [0.12, 0.64], *P* = 0.0041. (C) Log10 distribution of relative growth with median and IQR for IS and IR clinical *M. tuberculosis* isolates before rifampicin treatment. Statistical comparisons between IS and IR were made by using Wilcoxon rank-sum test.

For correlating relative growth in absence of antibiotics, we removed 19 outliers (supplementary figure 3 with 19 outliers), Intriguingly, slower growth before rifampicin treatment did not had significant correlation with higher growth fitness in rifampicin survival fraction at 15 days incubation in IS isolates (figure 4A regression coefficient −0.21, 95%CI [−0.44, 0.007], *P*=0.058). By contrast, correlation of slower growth with lower growth fitness in the long recovery period was observed in both IS and IR isolates (figure 4B, regression coefficient for IS=0.38 [0.15, 0.61], *P*=0.0014, and IR=0.38 [0.12, 0.64], *P*=0.0041). Comparing IS and IR isolates, IR isolates had slower growth in the absence of antibiotics (figure 4C, P<0.0001). Thus, slow growth before rifampicin treatment correlates with reduced growth fitness in certain rifampicin tolerant populations at 60 days incubation.

In case of IS isolates, higher rifampicin MICs correlated with lower rifampicin tolerance at long recovery period, 15 (−0.24 [−0.50, 0.022], *P*=0.073) and 60 days incubation (−0.31 [−0.53, −0.083], *P*=0.007, supplementary figure 4A), whereas IR isolates did not show such a negative correlation of rifampicin tolerance with rifampicin MICs (0.14 [−0.14, 0.41], *P*=0.33 and 0.21 [−0.057, 0.48], *P*=0.12, supplementary figure 4A). This latter observation might be due to increased growth fitness of IR rifampicin tolerant populations. In addition, there was no significant difference in rifampicin MICs distribution between IS and IR isolates (supplementary figure 4B).

We next investigated the association between isoniazid-resistant mutations in *M. tuberculosis* isolates and rifampicin tolerance distribution. These isolates had three different isoniazid-resistant mutations, *katG*_S315X (n=71), *inhA*_I21T (n=2) and fabG1_C-15X (n=6) and data not available for 5 isolates (supplementary figure 5). Due to low number of isolates with inhA and fabG1 mutations, it was not possible to identify the difference in rifampicin tolerance distribution between the isolates with different isoniazid-resistant mutations. Nevertheless, we observed wide distribution of rifampicin tolerance in isoniazid-resistant isolates with katG_S315X mutation itself (supplementary figure 5), indicating the role of other genetic or epi-genetic determinants influencing rifampicin tolerance.

### Higher rifampicin tolerance and growth fitness is associated with IR isolates from intensive phase of treatment with rifampicin

**Figure 5.**
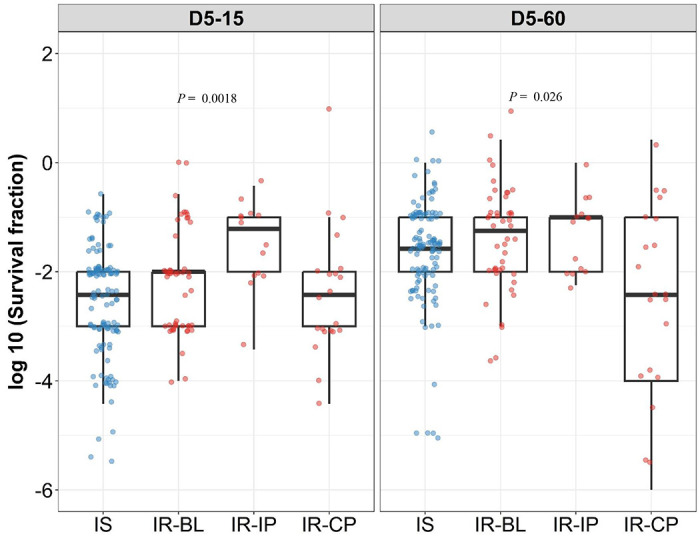
Rifampicin survival fraction distribution in isoniazid susceptible and longitudinal isoniazid resistant clinical *M. tuberculosis* isolates. Log10 rifampicin survival fraction distribution, with median and IQR (inter quartile range), of individual isoniazid susceptible (IS, blue dots, n = 119, 117 for D5-15, and D5-60 respectively), and longitudinal isoniazid resistant (IR, red dots, n = 84, 80 for D5-15, D5-60 respectively) isolates for 5 days of rifampicin treatment as determined at 15 and 60 days of incubation (D5-15, D5-60 respectively) grouped based on collection time as baseline (IR-BL, n = 49), intensive phase (IR-IP, n = 14), and continuous phase and relapse (IR-CP, n = 21). Statistical comparisons between groups were made by using Krusal-Walis test.

The IS isolates were collected only at baseline before treatment, whereas the IR isolates in our study were collected longitudinally from patients at different stages of treatment. The antibiotic treatment may select different *M. tuberculosis* genetic microvariants in the patients and lead to differences in rifampicin tolerance between longitudinal isolates. Therefore, we analyzed the rifampicin tolerance distribution in the IR isolates in three sub-groups, before treatment (IR-BL), initial two months of intensive phase of treatment with rifampicin in the regimen (IR-IP), continuous phase and relapse lacking rifampicin and any other antibiotics treatment selection respectively (IR-CP) (Figure 5). This grouping reflects TB-treatment regimen in Vietnam during the study period with rifampicin only in the initial two months of treatment^24^.

Interestingly, we observed significantly higher rifampicin tolerance and growth fitness in IR-IP group (*P*=0.0018, Figure 5 as compared to IS, IR-BL and IR-CP groups during 15 days recovery, indicating rifampicin treatment itself as a possible mechanism leading to the selection of *M. tuberculosis* tolerant microvariants in patients^17^.

**Figure 6.**
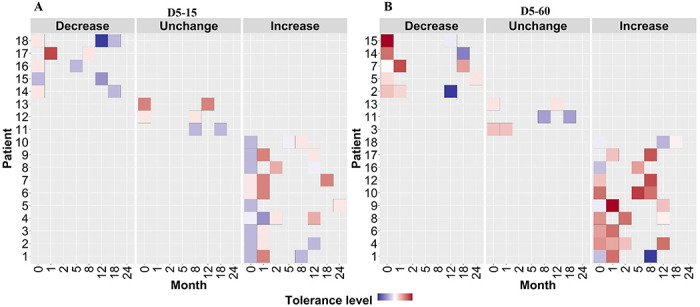
Rifampicin tolerance of longitudinal isoniazid resistant clinical *M. tuberculosis* isolates from individual patients. **(A, B)** Rifampicin tolerance heat map after 5 days of rifampicin treatment as determined at 15 and 60 days of incubation (D5-15, D5-60 respectively), of longitudinal isoniazid resistant clinical *M. tuberculosis* isolates collected from individual patients during different months of treatment and follow-up. Longitudinal isoniazid resistant clinical *M. tuberculosis* isolates from individual patients are grouped based on changes in rifampicin tolerance compared between initial and subsequent months of collection as decrease, un change and increase. Months (0 – 24) represent the different months the isolates were collected from patients during 8 months treatment and 24 months follow-up.

**Figure 7.**
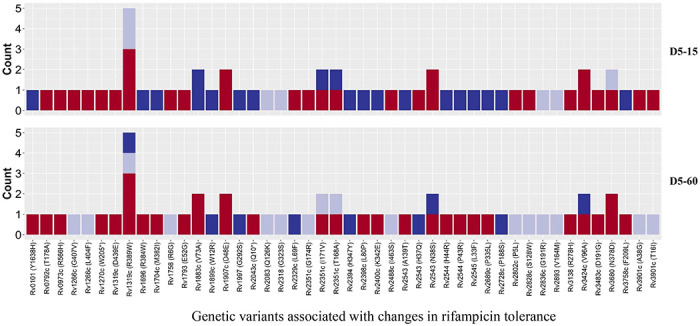
Non-synonymous single nucleotide polymorphism emerging in pair-wise comparison of longitudinally collected isoniazid resistant *M. tuberculosis* isolates from same patient associated with increase (red), decrease (dark blue) and no change (light violet) in rifampicin tolerance phenotype at 15 and 60 days of incubation (D5-15 and D5-60 respectively). Each count represent a single independent SNP emergence event.

To verify this finding, we grouped individual patients based on changes in rifampicin tolerance between their initial and subsequent IR isolates as decrease, unchanged and increase (Figure 6) and analyzed the difference in non-synonymous SNPs between the isolates from the same patients associated with differences in rifampicin tolerance (Figure 7, supplementary table 2). The SNPs difference between the longitudinally collected *M. tuberculosis* isolates from same patient were 0-3 except in one case (SNPs=11), indicating de-novo emergence or selection of genetic microvariants within the patient (supplementary table 2). Next, we analyzed the non-synonymous SNPs associated with the changes in rifampicin tolerance both at 15 and 60 days incubation. This included both genetic variants emerging as more than 90% of WGS reads and less-than 90% threshold used as a cut-off for calling SNPs. Several genes Rv0792c, Rv1266c, Rv1696, Rv1758, Rv1997, Rv2043c, Rv2329c, Rv2394, Rv2398c, Rv2400c, Rv2488c, Rv2545, Rv2689c, Rv3138, Rv3680 and Rv3758c previously reported to be associated with persistence, tolerance and survival within host had non-synonymous SNPs associated with changes in rifampicin tolerance (Figure 7, supplementary table 3 with references). This indicates mutations in multiple genes might affect rifampicin tolerance and growth fitness, since there was no one gene or genetic variant in *M. tuberculosis* in multiple patients consistently associated with increased or decreased rifampicin tolerance, or that mutations may be epistatic with the genetic background of the strain.

## Discussion

We investigated rifampicin tolerance in a large number of clinical isolates of *M. tuberculosis*. Overall clinical *M. tuberculosis* isolates showed higher levels of rifampicin tolerance than lab isolates as the average survival fraction post-rifampicin treatment decreased only by 90 to 99% over 5 days. Therefore, emergence of tolerance may mask persistent sub-populations or one assay may not capture both tolerance and persistence. We found that levels of rifampicin tolerance are widely distributed among isolates, with some genetically susceptible strains having similar susceptibility to rifampicin-mediated killing as *bona fide* rifampicin-resistant isolates, at least during the 5 days rifampicin exposure of our assay condition. Furthermore, IR isolates were more likely to harbor fast-growing subpopulations with high levels of rifampicin tolerance.

In our experimental set-up, we decided to assay the recovery of *M. tuberculosis* following rifampicin treatment at two distinct time-points, 15 and 60 days. Heterogeneity in regrowth following stress has been linked to a tradeoff between growth fitness and survival^26^, and it is likely that in *M. tuberculosis* such diversification in growth rates among rifampicin-tolerant subpopulations represents a balance between growth and persistence under antibiotic stress. A better molecular mechanistic understanding of drivers of growth-rate heterogeneity in *M. tuberculosis* may contribute to understanding the dynamics and drivers of tuberculosis relapse following standard therapy.

We also observed a variation in growth rate in the absence of antibiotic therapy. IR isolates were slower growing than IS isolates, which likely represents a fitness cost due to isoniazid-resistance-causing mutations and strain genetic background^31^. As expected, IS isolates, with slower growth in the absence of drug had a weak association with high levels of rifampicin tolerance at the 15 day time point (representing rapidly growing recovered cells), whereas both IS and IR isolates with slower growth in the absence of drug were significantly associated with lesser rifampicin survival fraction levels at 60 days– representing slow growing rifampicin tolerant bacilli. These data suggest that slower growth (in absence of drug) in both isoniazid susceptible and resistant isolates, perhaps due to fitness cost of mutations^31^, may be associated with more persister-like tolerant subpopulations.

By contrast, the association between rifampicin MIC and rifampicin tolerance showed a contrasting trend with isoniazid susceptibility. IS isolates showed decreased tolerance with increase in rifampicin MIC, but IR isolates did not show this association. This may indicate higher growth fitness of IR with rifampicin tolerance. Another important finding from our study is the emergence of higher rifampicin tolerance and growth fitness in longitudinal IR isolates under rifampicin treatment selection. This further supports the findings that multiple genetic microvariants may co-exist in patient and rapidly change their proportion under selection from host stresses and antibiotic treatment^32^. We also observed non-synonymous mutations in multiple genes, associated with persistence and host survival enriched with changes in rifampicin tolerance between the longitudinal isolates (supplementary table 3 with references). However, lack of convergent SNPs in the samples may be due to the relatively small sample size, interaction between SNPs and strain background or indication of a larger set of tolerance-related genes that independently affect bacterial growth and antibiotic tolerance^3^.

In addition to demonstrating a wide distribution of rifampicin tolerance among clinical isolates and the specific finding that IR isolates are associated with high-level rifampicin tolerance and growth fitness, our study reveals novel aspects of rifampicin tolerance associated with isoniazid susceptibility. Rifampicin treatment itself led to the selection of IR *M. tuberculosis* genetic microvariants with high rifampicin tolerance and increased growth fitness in patients. The precise mechanisms underlying these phenotypes will require further investigation, but it is intriguing to note that different *M. tuberculosis* lineages have varying liabilities for development of isoniazid resistance^33^, suggesting that clinical isolates may evolve diverse paths towards phenotypic drug resistance that impact fundamental bacterial physiology and tolerance to other antibiotics.

The wide range of observed rifampicin tolerance, spanning many orders of magnitude confirms findings of experimentally evolved drug tolerance to the laboratory isolate *M. tuberculosis*-H37Rv^10^ and extends our prior findings from a smaller-scale pilot study^28^. Given that almost all rifampicin resistance is via mutations in *rpoB*^34^, our findings suggest that first-line molecular testing for rifampicin susceptibility, which is replacing phenotypic drug susceptibility^35^, may not fully capture response to therapy. It needs to be further validated if these strains that are ‘hyper-tolerant’ to rifampicin are risk factors for poor clinical outcomes in IR-TB^24^.

An important observation arising from our study is that IR is associated with increased likelihood of high levels of tolerance to rifampicin – but only in faster growing recovered bacteria. Given the association of IR with emergence of rifampicin resistance^25^, our findings suggest a plausible mechanism by which isoniazid resistance, via rifampicin tolerance, acts as a ‘stepping stone’ to rifampicin resistance. The association between IR and rifampicin tolerance only held for fast-growing recovered bacteria. Given the observation that ‘growing’ rifampicin tolerant bacteria are over-represented after initiation of drug therapy in humans due to the specific regulation of *rpoB* in mycobacteria in response to rifampicin exposure^17^, this may represent a divergence between growing and non-replicating persister forms of antibiotic tolerance. A better understanding of which forms of tolerance contribute to clinically relevant response to therapy will be critical for tailoring individualized regimens for TB or improving treatment regimen for IR-TB^36^.

Our study has some limitations. We only assayed rifampicin tolerance under one standard axenic culture condition. It is known that antibiotic tolerance phenotypes vary considerably according to culture conditions^11^, with some phenotypes only emerging *in vitro* with specialized media, e.g. containing odd-chained fatty acids^11^. Secondly, contributors to antibiotic tolerance can be genetic, epigenetic or transient^9–12^, and there is considerable epistasis between genetic variation and antibiotic susceptibility. Our assay will not be able to capture drivers of tolerance that have been lost in the collection, banking, freezing and reviving of the *M. tuberculosis* isolates. Finally, the isolates were from a previous study^24^, and during the study period the old 8-month TB treatment regimen lacked rifampicin in the continuation phase^24^.

This study also reveals interesting aspects like fast and slow growing sub-populations and possible variation in lag-time distribution among clinical *M. tuberculosis* isolates. There can also be different mechanisms of tolerance and persistence among *M. tuberculosis* isolates, detailed investigations are required to further understand these aspects and its clinical relevance.

In conclusion, our study identifies a significant association between isoniazid-resistance and rifampicin tolerance in clinical isolates of *M. tuberculosis*. Our findings have implications for the requirement to consider heterogeneity in bacterial responses to antibiotics and emergence of antibiotic tolerant bacterial genetic microvariants in determining optimal tuberculosis treatment regimens.

## Methods

### Ethical approval

*M. tuberculosis* isolates in this study were a part of collection from a previous study^24^, approved by the Institutional Research Board of Pham Ngoc Thach Hospital as the supervisory institution of the district TB Units (DTUs) in southern Vietnam, Ho Chi Minh City Health Services and the Oxford University Tropical Research Ethics Committee (Oxtrec 030–07).

### Bacterial isolates

242 *M. tuberculosis* isolates, collected for a previous study in Vietnam were used in this study^24^. All the isolates were cultured in the biosafety level-3 laboratory at the Oxford University Clinical Research Unit, Ho Chi Minh city, Vietnam^24^.

### Rifampicin killing assay

Most-probable number-based rifampicin killing assay was done for the clinical *M. tuberculosis* isolates as per the published protocol^28^. *M. tuberculosis* isolates, after single subculture from archive, were inoculated in 7H9T medium (Middlebrook 7H9 broth supplemented with 0.2% glycerol, 10% OADC and 0.05% Tween-80) and incubated at 37^0^C until exponential phase with OD_600_ range of 0.4–0.6 is reached. All cultures were homogenized by vortexing for three minutes with sterile glass beads and diluted to the OD_600_ of 0.4. The diluted culture was used for measuring initial viable bacterial number by most probable number (MPN) method, using 96 well plates according to the published protocol^28^. Briefly the protocol was as follows, a 1 mL aliquot of *M. tuberculosis* culture was harvested, and the cell pellet was washed once. This washed culture was resuspended in 1mL culture and 100 μL was transferred to 96-well plates as an undiluted culture in duplicate for serial dilution. The undiluted culture was used for 10-fold serial dilution up to 10^9^ dilutions in microtiter plates. Immediately, after sampling for initial MPN (day 0), the remaining culture in the tube was treated with rifampicin (Merck-Sigma Aldrich, USA) at a final concentration of 2 μg/mL and incubated. On 2 and 5 days post-rifampicin treatment, viable bacterial number was determined again by MPN method as previously mentioned^28^. The growth in 96 well plate was recorded as images by the Vizion image system (Thermo Fisher, Scientific Inc, USA) after 15 and 60 days of incubation, beyond 60 days drying of plates were observed. The number of wells with visible bacterial growth was determined by two independent readings from two individuals, discrepancies between the two readings were verified and corrected by a third person reading. MPN value was calculated as mean MPN/mL. The survival fraction at 2 and 5 days post rifampicin treatment was calculated as compared to the initial MPN taken as 100% survival.

### Relative growth difference calculation from MPN number

For calculating relative growth difference of isolates before rifampicin treatment, the log10 MPN ratio between 15 and 60 days of incubation were taken to determine the relative proportion of fast and slow growing sub-population. A log_10_ MPN ratio close to 0 indicated less growth heterogeneity in the population, whereas a ratio less than 0 indicated presence of growth heterogeneity due to the presence of fast and slow growing, or heterogeneity in the lag time distribution of sub-populations.

### Drug susceptibility testing

Microtiter drug susceptibility testing was performed using UKMYC6 plates (Thermo Fisher, Scientific Inc., USA) for determining initial rifampicin and isoniazid phenotypic susceptibility^37^. Briefly, three weeks-old *M. tuberculosis* colonies from Lowenstein-Jensen medium were used to make cellular suspension in 10 mL saline-Tween80 tube with glass beads (Thermo Fisher, Scientific Inc., USA) and adjusted to 0.5 McFarland units. The suspension is diluted in 7H9 broth (Thermo Fisher, Scientific Inc., USA) and inoculated into 96-well microtiter plate using a semi-automated Sensititre Autoinoculator (Thermo Fisher, Scientific Inc., USA). Plates were sealed with plastic sheet and incubated at 37^0^C for 14 to 21 days. The minimum inhibitory concentration (MIC) was measured by a Sensititre Vizion Digital MIC Viewing system (Thermo Fisher, Scientific Inc., USA) and considered valid if there was growth in the drug free control wells. The clinical resistant cut-off concentrations for isoniazid and rifampicin were 0.1 and 1 μg/mL, respectively.

The IR isolates were also confirmed using BACTEC MGIT 960 SIRE Kit (Becton Dickinson) according to the manufacturer’s instruction in the biosafety level-3 laboratory at the Oxford University Clinical Research Unit^24^. Phenotypic DST was done for streptomycin (1.0 μg/mL), isoniazid (0.1 μg/mL), rifampicin (1.0 μg/mL) and ethambutol (5.0 μg/mL)^24^. Whole genome sequence data was available for the isolates from previously published study^25^ and the Mykrobe predictor TB software platform was used for genotypic antibiotic susceptibility determination of *M. tuberculosis* isolates^38^.

### Statistical analysis

MDK90 values, and its credible interval was estimated using a linear mixed effect model with a Bayesian approach (brm function, brms package).We used the linear mixed effect model for survival analysis as the data consists of repeated measurements at specific time points. For the linear mixed effect model with the bacterial strains as a random effect, we use the Bayesian approach with non-informative priors, which is equivalent to the frequentist approach. The fixed effect relates to the explanatory variable we are utilizing to predict the outcome. Specifically, our outcome measure is the log_10_ survival fraction. The explanatory variables encompass isoniazid susceptibility (categorized as isoniazid susceptible or resistant), the day of sample collection (0, 2, and 5 days), and the duration of incubation (15, and 60 days).

Wilcoxon rank-sum test (stat_compare_means function, ggpubr package) was used to test the null hypothesis that the IS and IR groups have the same continuous distribution, as it is a non-parametric test that does not require a strong assumption about the normality of the distribution of the variable. Chi-Square test (odds ratio function, epitools package) was used to determine if there is a significant relationship between IR and rifampicin tolerance. Cochran Armitage test (CochranArmitageTest function, DescTools package) was performed to test for trend in IR proportion across the levels of rifampicin tolerance. Linear regression (lm function, stats package) was used to evaluate the correlation between rifampicin survival fraction and relative growth.

Statistical analyses and graphs were plotted using R, version 4·0·1^39^ and p-values of ≤0·05 were considered statistically significant.

### MDK_90, 99_ and _99.99_ calculation

In addition to MDK90 calculated by linear mixed effect model, we also determined the MDK values at 90, 99 and 99.99% reduction in survival fractions for all the *M. tuberculosis* isolates by the following method. The log_10_ MPN values at Day0, Day 2, and Day 5 were used to calculate the respective MDK time for 90%, 99%, and 99.99% reduction in fraction of survival. The calculation of MDK time for individual isolate was based on modelling kill curve as two similar triangles and using the basic proportionality theorem as shown in the flow chart (Supplementary figure 6) to determine the different length of X-axis (Days post rifampicin treatment) corresponding to decline in survival fraction in Y-axis for each MDK time (MDK_90, 99_ and _99.99_).

For example, in case of MDK90, Y0 (MPN number at day 0), Y2 ((MPN number at day 2), and Y5 ((MPN number at day 5).

First condition tested is, if 90% reduction in survival fraction happened before or at day 2 by checking if log_10_ MPN number at day 2 is less than or equal to 90% reduction as compared to Y0. If the condition is true then the MDK is calculated as x-axis length DF in the two similar triangles modelled in A (triangles ACB and AFD) and corresponding formula for X given below. If the first condition is false then two similar triangles are modelled as in B (triangles ABC and DEC) and X is calculated as 5 – EC. Similarly, for MDK_99_ and MDK_99.99_ time are calculated by applying the condition for 99% and 99.99% reduction in survival fraction.

### Single nucleotide polymorphism difference between longitudinal isoniazid-resistant isolates with differences in rifampicin tolerance

We used whole genome sequence data and genetic variants analysis previously published for identifying non-synonymous single nucleotide polymorphisms (SNPs) emerging in longitudinal isolates from same patients associated with changes in rifampicin tolerance between the isolates^25^.

## Figures and Tables

**Table 1.** Association of rifampicin tolerance level with isoniazid susceptibility

Incubation time	Rifampicin tolerance level	Isoniazid Susceptible (N = 119)	Isoniazid Resistant (N = 84)	P	OR (95%CI)	P trend
D5-15	Low tolerance (N, %)	26 (79, 26/33)	7 (21, 7/33)			0.0038
	Medium tolerance (N, %)	72 (58, 72/124)	52 (42, 52/124)	0.029	2.68 (1.08-6.65)	
	High tolerance (N, %)	21 (46, 21/46)	25 (54, 25/46)	0.003	4.42 (1.60-12.22)	

D5-60	Low tolerance (N, %)	26 (55, 26/47)	21 (45, 21/47)			0.67
	Medium tolerance (N, %)	74 (64, 74/115)	41 (36, 41/115)	0.28	0.69 (0.34-1.37)	
	High tolerance (N, %)	17 (49, 17/35)	18 (51, 18/35)	0.55	1.31 (0.55-3.15)	

N = number of isolates. (% as percentage, N/total number (IS + IR)

P = P-value determined using Chi-square test.

P trend = P-value determined using Cochran-Armitage test.

OR = odds ratio.

95%CI = 95% confidence interval.
